# Beamline K11 DIAD: a new instrument for dual imaging and diffraction at Diamond Light Source

**DOI:** 10.1107/S1600577521009875

**Published:** 2021-10-22

**Authors:** Christina Reinhard, Michael Drakopoulos, Sharif I. Ahmed, Hans Deyhle, Andrew James, Christopher M. Charlesworth, Martin Burt, John Sutter, Steven Alexander, Peter Garland, Thomas Yates, Russell Marshall, Ben Kemp, Edmund Warrick, Armando Pueyos, Ben Bradnick, Maurizio Nagni, A. Douglas Winter, Jacob Filik, Mark Basham, Nicola Wadeson, Oliver N. F. King, Navid Aslani, Andrew J. Dent

**Affiliations:** a Diamond Light Source, Harwell Science and Innovation Campus, Didcot, Oxfordshire OX11 0DE, United Kingdom; b NSLS-II, Brookhaven National Laboratory, Upton, NY 11973, USA; c Rosalind Franklin Institute, Harwell Science and Innovation Campus, Didcot, Oxfordshire OX11 0FA, United Kingdom

**Keywords:** synchrotron, full-field micro-tomography, micro-diffraction, time-resolved studies

## Abstract

The Dual Imaging and Diffraction (DIAD) beamline at Diamond Light Source is a new dual-beam instrument for full-field imaging/tomography and powder diffraction. This instrument provides the user community with the capability to dynamically image 2D and 3D complex structures and perform phase identification and/or strain mapping using micro-diffraction. The aim is to enable *in situ* and *in operando* experiments that require spatially correlated results from both techniques, by providing measurements from the same specimen location quasi-simultaneously. Using an unusual optical layout, DIAD has two independent beams originating from one source that operate in the medium energy range (7–38 keV) and are combined at one sample position. Here, either radiography or tomography can be performed using monochromatic or pink beam, with a 1.4 mm × 1.2 mm field of view and a feature resolution of 1.2 µm. Micro-diffraction is possible with a variable beam size between 13 µm × 4 µm and 50 µm × 50 µm. One key functionality of the beamline is image-guided diffraction, a setup in which the micro-diffraction beam can be scanned over the complete area of the imaging field-of-view. This moving beam setup enables the collection of location-specific information about the phase composition and/or strains at any given position within the image/tomography field of view. The dual beam design allows fast switching between imaging and diffraction mode without the need of complicated and time-consuming mode switches. Real-time selection of areas of interest for diffraction measurements as well as the simultaneous collection of both imaging and diffraction data of (irreversible) *in situ* and *in operando* experiments are possible.

## Abstract 2

This manuscript summarizes the capabilities of the new DIAD beamline for dual imaging and diffraction (DIAD) at Diamond Light Source, dedicated to quasi-simultaneous, time-resolved imaging/tomography and powder diffraction for 3D microstructure and phase identification/strain analysis studies.

## 1. Introduction

To fully understand the properties of many materials, there is a scientific need to understand the 3D microstructure, the phase composition and the stress distribution of the material. Examples include corrosion, where growth kinetics are determined by the shape of a corrosion pit as well as the presence of (also intermediate) corrosion products (Noell *et al.*, 2020 ▸); the mechanical stability of bone, where fracture depends on the local bone microstructure, the presence of micro-cracks and the local stress distribution around these cracks (Ma *et al.*, 2016 ▸); and the discharging of batteries, where fracture due to repeated stress and strain during the battery charging/discharging cycles leads to premature failure (Llewellyn *et al.*, 2020 ▸).

Therefore, new tools are needed to provide simultaneous information about the 3D microstructure, the phases present and/or the local stress distribution. The 3D microstructure of materials can be determined from X-ray imaging and tomography experiments; the phase composition and stress distribution can be established from X-ray diffraction techniques. Many synchrotron beamlines provide the tools to investigate either one or the other, such as the imaging beamline I13 (Rau, 2017 ▸) and the powder diffraction beamline I11 (Thompson *et al.*, 2009 ▸) at Diamond Light Source. Some beamlines can provide both imaging and diffraction (Drakopoulos *et al.*, 2015 ▸; King *et al.*, 2016 ▸; Schell *et al.*, 2013 ▸), but not at once. These beamlines require changes between setups that are often complicated and time-consuming, thus limiting the ability to examine the evolution through time at a given location in a specimen.

The Dual Imaging and Diffraction (DIAD) beamline is a new facility at Diamond Light Source that provides both imaging and diffraction capabilities in one instrument. Its novel dual beam design makes the mode switches required at other beamlines unnecessary. The beamline operates with two independent beams meeting at the sample position, and with two detector setups acquiring either a full-field radiograph or a diffraction pattern. The switching frequency between these two acquisition modes can be as high as a few Hz.

The beamline enables *in situ* and *in operando* measurements and, combined with automated data processing, auto-segmentation for tomography data and diffraction data reduction, giving users the opportunity to carry out fast decision making.

In the following sections, we describe the beamline setup and highlight some early commissioning results and their potential benefit to the wider user community. Additional information can be found on the DIAD website (https://www.diamond.ac.uk/Instruments/Imaging-and-Microscopy/DIAD.html).

## 2. X-ray source and optics

**Figure 1:**
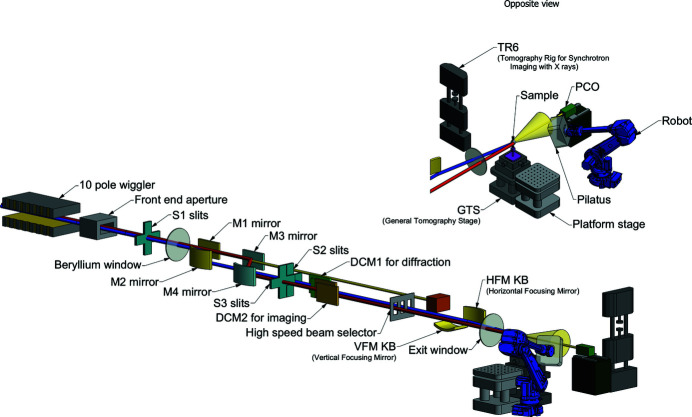
Schematic of the DIAD beamline including X-ray optics and endstation. The blue ray represents the diffraction and the red ray the imaging beam.

DIAD has two independent branches for imaging and diffraction, sharing one wiggler source. The beamline optics for each branch are shown in Fig. 1 ▸ and Table 1 ▸.

The DIAD wiggler is a fixed-gap hybrid device (Martin *et al.*, 2019 ▸) with a period of 116 mm, a peak field of 1.54 T and a total length of 0.7 m. The wiggler is permanently in position but can be manually removed from the ring if required.

The beamline has an innovative optics layout that allows two independent beams to run in parallel to the sample position. We are not aware of a similar setup being used elsewhere.

A beryllium window acts as a separation between the storage ring and beamline vacuum.

The full wiggler beam is split into two independent beams at the first mirror M1 by inserting the mirror partially into the wiggler fan and reflecting the part of the beam for the diffraction branch onto mirror M2. The part of the wiggler fan passing M1 is utilized for the imaging beam by reflecting from mirror M3 to mirror M4.

M1 and M3 are flat mirrors whereas M2 and M4 collimate the beams creating a parallel beam setup. All four mirrors are designed with three stripes for harmonic rejection, which is of particular importance for the diffraction beam. The Si stripe is used for operation below 14 keV, the Cr stripe in the range from 14 keV to 22 keV, and the Pt stripe for energies above 22 keV.

The double-crystal monochromators (DCM) DCM1 and DCM2 operate in the energy range 7–38 keV, with cryo-cooled Si (111) double-bounce crystals. The energy for each DCM can be chosen independently.

**Figure 2:**
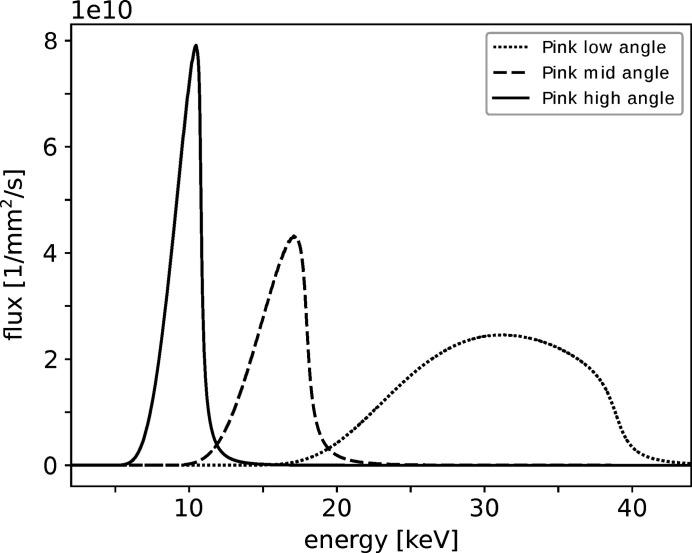
Simulated spectra of the photon flux for pink beam imaging at the sample position: the energy of the photon flux can be adapted by using a series of different mirror and filter settings (low angle: M3/M4 angles 2.1 mrad, Pt stripe, 4 mm Al; mid angle: M3/M4 angles 2.5 mrad, Cr stripe, 2 mm Al; high angle: M3/M4 angles 2.9 mrad, Cr stripe, 0.1 mm Al).

For pink beam imaging, the photon energy is set by a series of mirror and filter parameters. The simulated flux for the pink imaging at the sample position is displayed in Fig. 2 ▸.

All beamline optics are in a horizontal bounce arrangement. Despite reducing the achievable flux at the sample position due to polarization losses below 10 keV, a horizontal optics arrangement was chosen for simplicity of keeping both beams in the same plane and allowing them to reach the sample position without being blocked by the optics of the other beam.

The beam selector intersects both beams. It is a rotary system of cams which allows the user to select either the imaging or the diffraction beam and blocks the other. This avoids cross-talk of the two X-ray signals during acquisition. Switching between beams at speeds of up to a few Hz is possible, enabling fast experiments.

Downstream of the beam selector, there are no further optics for the imaging beam. The diffraction beam, in contrast, has a Kirkpatrick–Baez (KB) mirror system to focus the beam and shine the diffraction beam onto the position of the imaging beam at the sample position.

## 3. Endstation

**Figure 3:**
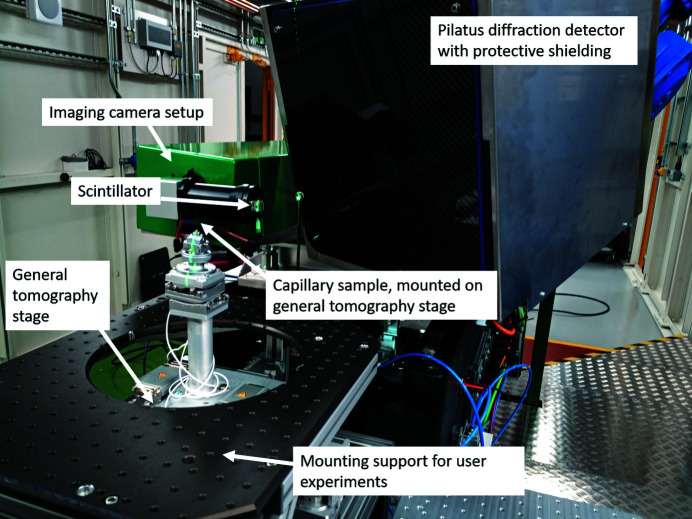
DIAD endstation: data are collected using the imaging camera setup for radiography/tomography and the Pilatus 2M CdTe diffraction detector for the diffraction pattern; a capillary specimen is mounted on top of the general tomography stage.

The DIAD endstation consists of multiple elements: (i) the specimen manipulation stages, (ii) the imaging camera setup, (iii) the diffraction camera setup and (iv) auxiliary equipment to support user experiments. The main endstation components are shown in Fig. 3 ▸.

### 3.1. Sample manipulation stages

DIAD has two sample manipulation stages for specimen mounting, moving on a shared base translation.

The General Tomography Stage (GTS) allows for horizontal and vertical translation of the specimen, as well as a rotation stage for tomographic data collection and is restricted to light-weight specimen and sample environments.

Heavy-specimen or sample environments up to 100 kg can be accommodated on the Platform Stage. This stage is suitable for the positioning of heavy samples, or sample environments, into the beam, including user-supplied equipment. The Platform Stage is restricted to radiography experiments.

A summary of the main stage parameters is given in Table 2 ▸.

### 3.2. Imaging camera setup

#### 3.2.1. Hardware

The imaging camera setup is used for micrometre-scale resolution radiography and tomography. The setup consists of a GGG:Eu or LuAG:Ce scintillator, optically coupled with custom-made radiation-hard optics from Sill Optics GmbH & Co KG to a PCO Edge 5.5 imaging camera sensor.

For optimal spatial resolution and light efficiency, the thickness of the scintillator is matched to the depth of focus of the optical system (Koch *et al.*, 1998 ▸).

The currently used PCO sensor is too small to cover the complete X-ray field of view of 1.7 mm × 1.7 mm. To preserve the resolution, we instead image an area of 1.4 mm × 1.2 mm, at a pixel size of 0.54 µm, at the specimen position. To image the complete X-ray beam, the imaging setup will be upgraded to an Andor Balor 17-F 12 which has a larger sensor at the same pixel resolution.

To facilitate both techniques to run quasi-simultaneously, the imaging camera must not block the diffracted photons from the specimen reaching the diffraction detector. To achieve this, the scintillator housing has been minimized and the imaging camera has been mounted in the horizontal, with the aim to keep the in-board side of the beamline clear for the diffraction setup. In addition, the sample-to-detector distance has been increased to 35 mm; hence, imaging will always have a contribution of in-line phase contrast via propagation distance, increasing contrast by edge enhancement (Fitzgerald, 2000 ▸).

#### 3.2.2. Image resolution

The image distortion and resolution limits of the new optics were assessed under X-ray conditions, using monochromatic beam at 15 keV.

Distortion was measured from a distortion test pattern (array of gold columns of 12 µm diameter on Si, 50 µm pitch in horizontal and vertical direction, column height 10 µm) across the whole image field of view. The optical system does not have geometrical distortion.

**Figure 4:**
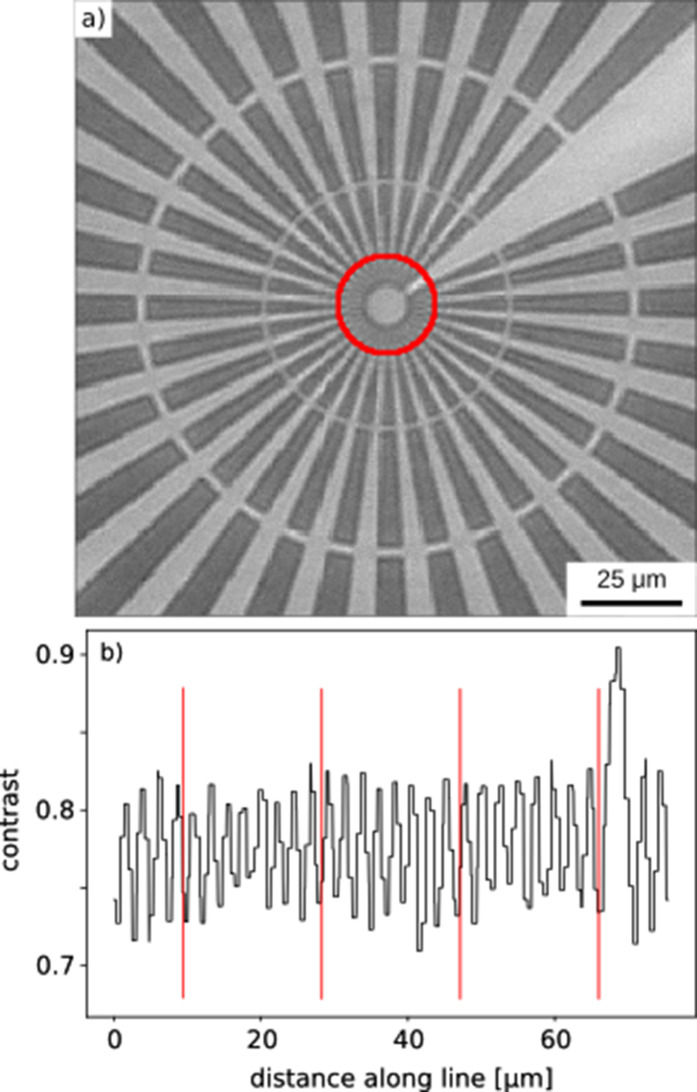
Features of 1.2 µm size can be resolved with the imaging camera setup. (*a*) High-resolution radiographic projection of a Siemens star (collected with monochromatic beam at 15 keV); the red circle highlights a radius of 12 µm around the Siemens star centre for pixel intensity measurement. (*b*) Pixel intensity along the red circle, with ∼8% difference between high- and low-contrast regions.

The image resolution was measured using an X-ray Siemens star pattern (gold deposited on Si, 1.6 µm thickness, line width from 0.5 µm to 75 µm), seen in Fig. 4 ▸. The imaging camera system can resolve features of 1.2 µm in size.

### 3.3. Diffraction setup

#### 3.3.1. Beam delivery

It is common for micro-diffraction beamlines to have a fixed diffraction beam and move the specimen. This has many advantages, the main one being a stable relative position of the beam/sample intersection and the detector, providing a very accurate transformation from pixel to reciprocal space. The disadvantage of this setup arises when the specimen is large, heavy or fragile and therefore cannot be moved rapidly to monitor dynamic processes in different regions of interest without damage. In such cases, it can actually be easier to shift the diffraction beam instead while the imaging system and the sample remain fixed. DIAD’s unique dual-beam setup permits this, allowing DIAD to monitor the dynamic processes in the specimen by using the imaging camera, identify interesting regions in the sample as they appear, and immediately point the diffraction beam to those regions to collect diffraction data from them in real time.

To deliver this capability, the focused beam for the diffraction branch is delivered from a Kirkpatrick–Baez (KB) mirror system. The two mirrors of the KB pair are identical in their optical parameters. They are 150 mm long, Pt-coated and work at a fixed incident angle of 2 mrad. They are bent to the required shape by two independent bending mechanisms at the ends of the mirror substrate for each mirror. The effective entrance aperture of the KB-mirror pair is 0.3 mm × 0.3 mm. The beam illuminating the KB is collimated by the upstream mirror M2 to a size of 2.1 mm × 2.1 mm. The KB mirrors are translated within this beam through a 1.7 mm × 1.7 mm area, the size of the imaging beam. Thus, they select different regions of the collimated incident beam for focusing onto correspondingly different regions of the sample. Furthermore, the beam size can be changed by varying the bending of the mirrors from as small as 13 µm × 4 µm (horizontal and vertical) to as large as 50 µm × 50 µm.

While advantageous for monitoring dynamic processes, the dual-beam mode with moving beam has its costs. Firstly, the relative position of the diffraction detector and beam/sample intersection is no longer fixed, so the calibration required to transform from pixel to reciprocal space is more complex. Secondly, the position of the imaging detector limits the ability to have the diffraction beam centre (and complete Debye–Scherrer rings) on the diffraction detector, making the determination of sample strain less precise than for experiments with a stationary beam and full diffraction rings. A more complete discussion on approaches to calibrating this moving beam configuration, and the limitations on strain resolution, are in preparation.

#### 3.3.2. Diffraction detection system

As a diffraction detector, the beamline is operating a Pilatus2 CdTe 2M hybrid detector (Dectris AG, Baden-Daetwill, Switzerland). The CdTe version was chosen over the Si version for its better sensitivity at higher energies.

**Figure 5:**
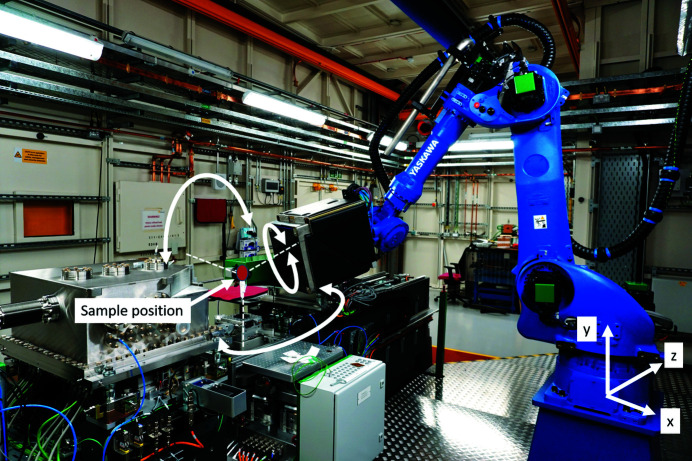
Industrial robot arm positioning Pilatus diffraction detector in a semi-hemisphere around the sample position.

Careful consideration of the diffraction geometry is required for all measurements, including the beam energy, the detector position and the required *q*-range of the experiment. To give the maximum flexibility of the detector positioning, the detector is mounted on an industrial robot arm (MOTOMAN M H225, Yaskawa Europe GmbH, Allerhausen, Germany), shown in Fig. 5 ▸. The robot facilitates detector positioning in the semi-hemisphere around the sample, with typical sample-to-detector distances between 300 and 600 mm.

Detector position stability over time is essential to collect good diffraction data. As the detector and the support structure have a total weight of 139 kg, the robot positioning was of particular concern and, hence, was assessed in a prior study, yielding that the detector position drifts less than 12 µm over a 12 h period (Reinhard *et al.*, 2021 ▸). For comparison, the pixel size of the diffraction detector is 172 µm.

Due to the positioning of the Pilatus detector off-centre relative to the incident beam in the vicinity of the imaging camera setup, only partial Debye–Scherer rings can be collected.

#### 3.3.3. Moving beam calibration

As mentioned in Section 3.3.1 (*Beam delivery*), most micro-diffraction beamlines have fixed beam and diffraction detector positions. Not only are these positions fixed, but beamlines are also designed such that these positions are as stable as possible, so that the limit of the pixel-to-reciprocal space transformation accuracy is the calibration.

To calibrate a diffraction experiment, a measurement is taken of a well characterized sample [usually a standard from the National Institute of Standards and Technology (NIST)], then the positions of the Debye–Scherrer rings are used to determine the position of the detector relative to the beam/sample intersection point and the precise energy of the X-rays. Multiple images may be required to determine both detector distance and energy at low scattering angle, for a high-energy beam (Hart *et al.*, 2013 ▸).

Once the calibration is complete, its correctness can be validated by refining the reduced data against the NIST standard lattice parameters and checking the deviation of the position of a reflection around the radius of the ring.

**Figure 6:**
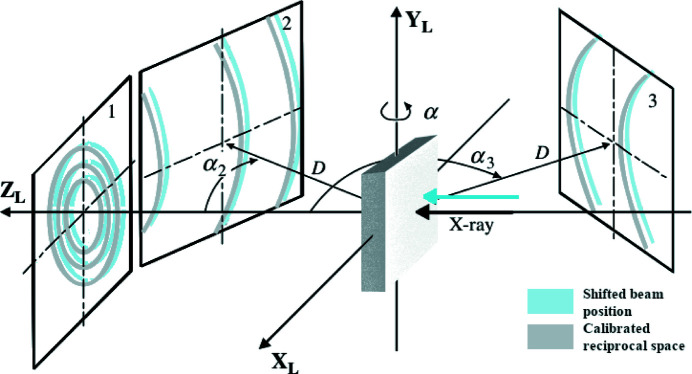
Diffraction geometry with three different detector positions [adapted and published with permission of Wiley from He (2018 ▸); permission conveyed through Copyright Clearance Center, Inc.]. As the incoming beam shifts relative to the detector, the centre of the diffraction cones shifts on the detector.

For DIAD, the detector is stationary relative to the sample and the beam moves in the Cartesian laboratory coordinate system. To determine the position and orientation of the detector relative to the moving beam, DIAD treats the beam as stationary and translates the entire laboratory coordinate system instead of the beam. A single calibration pattern is taken with the diffraction probe located at the position where the centre of the imaging field of view (FOV) would be if the scintillator was positioned at the sample position. From this single measurement, it is possible to determine the parameters required to convert pixel-to-reciprocal space for that location, but not to uniquely determine the full orientation of the detector. The use of un-textured powders (typical of NIST standards) leads to different combinations of detector tilt and rotation producing equivalent conic sections. Hence, when extrapolating translations of the diffraction probe position, the calibrated orientation of the detector is important. To approximate the full detector orientation, the beam is moved across the full FOV of the powder standard in the imaging camera taking several diffraction patterns (typically nine), see Fig. 6 ▸. This second calibration allows the orientation of the detector relative to specimen and beam translations to be determined. After this process the calibrated detector position and orientation can be used to convert pixel to reciprocal space for any position of the KB system.

Calibration of this type of setup is a multi-stage process but, once calibrated, the calibration parameters remain valid until a change of monochromator energy, detector position or beam size on the diffraction branch occurs. The calibration process will be described more fully elsewhere.

**Figure 7:**
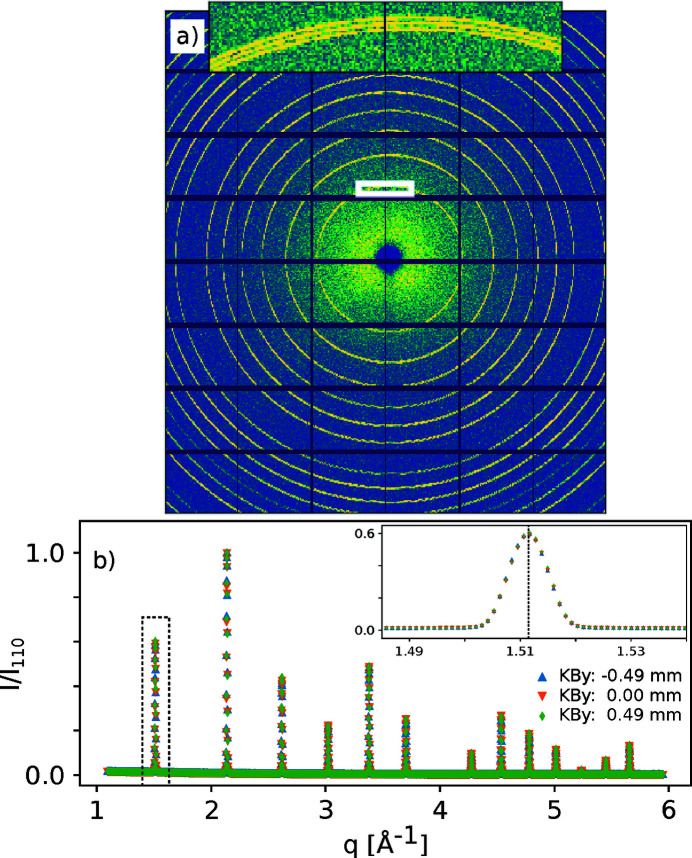
LaB_6_ calibration pattern collected at three vertical locations of a 0.3 mm-diameter capillary during rotation, at an incident beam energy of 24.69 keV. The sum of the three raw images is shown in (*a*) with the inset (white) showing a region of the (100) reflection at a higher magnification. The azimuthally integrated patterns are shown in (*b*). The intensities have been scaled relative to the maximum value obtained for the (110) reflection for each pattern. The inset shows the (100) peak in more detail including the expected position for a *d*-spacing of 4.15692 Å. The alignment across all locations shows that the automated calibration procedure compensating for the moving beam is effective and instrumental artefacts can be corrected for.

Data reduction steps that compensate the moving beam offsets have been implemented in *Data Analysis WorkbeNch* (*DAWN*) (Basham *et al.*, 2015 ▸; Filik *et al.*, 2017 ▸), used extensively at Diamond; hence live data reduction is possible. An example of data collected from three vertical positions in an LaB_6_ (NIST SRM660b) capillary, collected at 24.69 keV, is shown in Fig. 7 ▸(*a*) as a single summed image. The three rings produced by summation show the beam movement and, after offset correction, the peak positions of all 1D patterns are visibly in the same position [Fig. 7 ▸(*b*)], independent of the location where the pattern was collected confirming the effectiveness of the calibration process.

### 3.4. Auxiliary equipment

DIAD staff are already working on dedicated sample environments for future *in situ* and *in operando* specimen processing.

To accommodate users performing experiments in a variety of environmental conditions, a controllable environment chamber was developed. The environmental cell provides control over the temperature (5–85°C) and humidity (5–95% RH) and will mainly be used for biological and biomedical research.

A third specimen manipulation area with the TR6 (TR SIX – Tomography Rig for Synchrotron Imaging with X-rays) mechanical test rig for tensile/compression experiments will be added to the endstation to provide integrated tomography capability. TR6 includes two rotation stages and an actuation system to allow compressive or tensile loads to be applied to the specimen while ensuring concentric rotation of the specimen to avoid asymmetric loading or unwanted forces. It can provide continuous rotation of the specimen around 360° whilst under load. Furthermore, the rig will be equipped with load cells to measure the axial load and a video extensometer to measure axial and transverse strain on the specimen.

## 4. Data acquisition

DIAD can independently collect X-ray radiographic and X-ray diffraction measurements which can be processed into tomograms and diffraction maps. These two X-ray techniques can be combined into a single workflow consisting of several modes of data acquisition: (i) radiography-guided diffraction, (ii) tomography-guided diffraction and (iii) fast interleaving of radiographs and diffraction spots. The *GDA* (*General Data Acquisition*) software coordinates all data acquisition tasks at the beamline, using a continuous scanning mechanism (Walton *et al.*, 2015 ▸). The beamline provides a sophisticated mechanism for automating the data acquisition with the ‘experimental protocol’, a framework that enables experiments to be controlled through hardware triggering and/or other specimen parameters.

### 4.1. Radiography-guided diffraction: point and shoot

**Figure 8:**
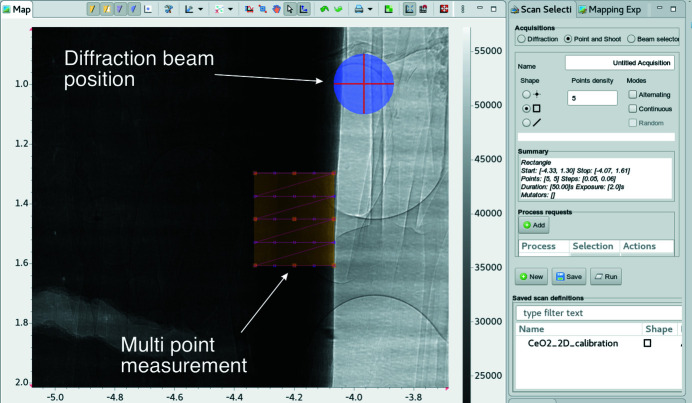
Point-and-shoot GUI for radiography-guided diffraction within the *GDA* software. The position of the diffraction beam is overlayed on the radiograph. Specimen: cortical mouse bone, imaging beam for radiography at 37 keV, diffraction beam at 18.5 keV.

Radiography-guided diffraction, also called point-and-shoot mode, enables the user to view a live radiograph of the specimen and visually identify a region (or point) of interest (ROI) for phase or strain analysis through micro-diffraction. With a graphical user interface (GUI), the users can select this location on screen, as seen in Fig. 8 ▸. The diffraction beam will move to this position and collect diffraction data from the chosen location. This is the basic mode of image-guided diffraction.

### 4.2. Tomography-guided diffraction: particle tracking

**Figure 9:**
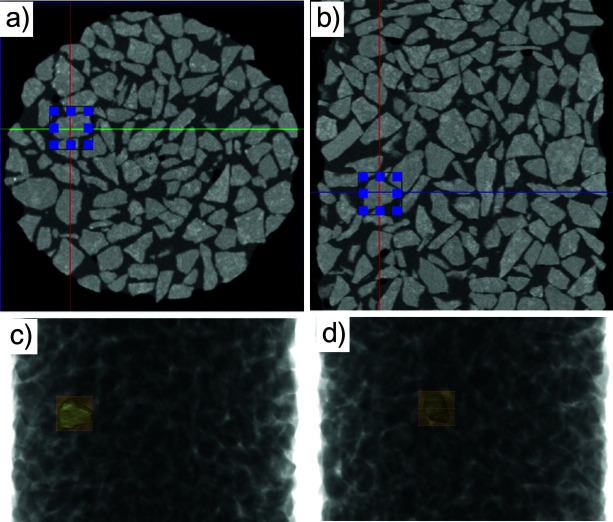
The particle-tracking GUI allows to highlight a cubic region of interest within the reconstructed 3D tomogram using an (*a*) horizontal and (*b*) vertical slice through the volume. Diffraction maps can then be collected at any number of rotation angles with the diffraction beam automatically tracking the region or particle, for example at (*c*) 0° and (*d*) 90°. Tomography data of a soil specimen [adapted from Ahmed, 2014 ▸)] is shown for illustration purposes – no actual data were collected at this point.

Tomography-guided diffraction, or particle tracking mode, aims to identify an area of interest within a 3D volume and is as such an extension of the point-and-shoot mode from 2D into 3D space. For particle tracking, 3D tomography data are used to guide the diffraction setup. The decision-making process for this mode involves multiple steps. Live processing with reconstruction, segmentation and visualization of the tomography data are all required as part of the decision-making process to aid the identification of an ROI. Once an ROI has been identified (see Fig. 9 ▸, conceptual view of a virtual sample), single or multiple diffraction patterns are acquired by moving the diffraction beam to the requested location, and possibly changing the rotation angle of the general tomography stage, thus enabling diffraction patterns of the ROI to be collected at multiple locations. This mode is still in the commissioning phase.

### 4.3. Fast interleaving: beam selector scan

Beam selector scans allow for rapid switching between the imaging and static diffraction beam at up to a few Hz. At the highest rate, one radiograph and one diffraction pattern can be collected, in sequence, every 200 ms, depending on specimen quality and scattering power. This mode is designed to investigate fast *in situ* processes that require both radiographs and diffraction data in rapid succession, *e.g.* phase transitions.

### 4.4. Experimental protocol

**Figure 10:**
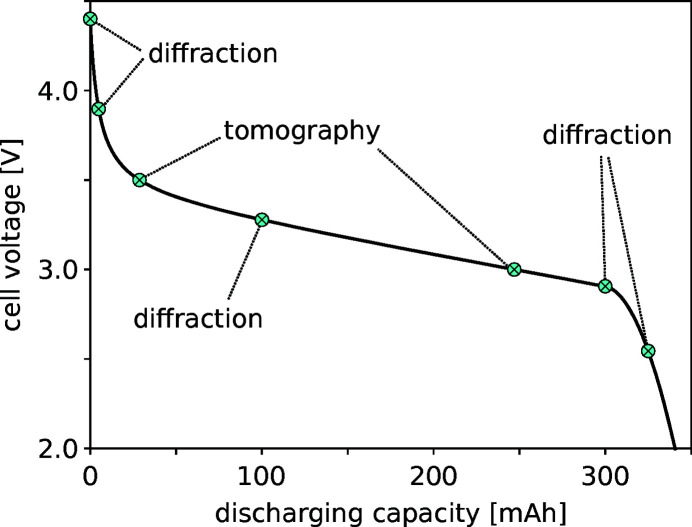
Example application of the experimental protocol. Trigger points, based on the conditions in Table 3 ▸, are displayed on the planned test path with a threshold for the trigger value to compensate for errors in sensor readout and the associated X-ray measurement.

The experimental protocol is a GUI tool that allows automatic collection of X-ray measurements for *in situ* experiments that utilize specimen processing auxiliary equipment, such as the TR6 or the potentiostat. Users pre-define measurement points or ‘triggers’ along the test path of the *in situ* process. Once the experiment is started, the system continually monitors the *in situ* process; when a trigger condition is met, the associated X-ray measurement is initiated. Triggers can be either temporal or based on an available sensor. For example, Table 3 ▸ shows the pre-defined trigger points for automatic data collection during a battery discharge experiment using a fully integrated potentiostat. Fig. 10 ▸ highlights a typical discharge–voltage plot when tomography and diffraction measurements are triggered.

### 4.5. Automatic post-processing

A dedicated post-processing pipeline for tomography and diffraction data analysis is available to quickly reconstruct and reduce the data.

Data post-processing tasks such as tomographic reconstruction, image segmentation and diffraction pattern reduction pose a significant challenge to users of synchrotron beamlines. Consequently, DIAD has implemented discrete pipelines for imaging and diffraction that automatically process X-ray measurements.

A Zocalo processing pipeline (Gerstel *et al.*, 2019 ▸) coordinates all the processing steps in various programs.

Standard experimental tomography data are processed without the need for live decision making, taking place as a multi-step process within the SAVU pipeline (Wadeson & Basham, 2016 ▸) using flat-field correction, centre-of-rotation finding (Vo *et al.*, 2014 ▸) and reconstruction (Van Aarle *et al.*, 2015 ▸). Where required, additional processing steps such as ring removal (Vo *et al.*, 2018 ▸) and/or Paganin phase retrieval (Paganin *et al.*, 2002 ▸) are used. For more advanced data processing requirements, automatic segmentation steps using *SuRVoS* (King *et al.*, 2016 ▸) can be added, leveraging pre-defined machine-learning models. Running the complete data processing pipeline is time consuming. Thus, for live data processing, the pipeline can be modified by adapting the processing steps (mostly by changing and/or removing steps) to decrease the post-processing time.

Diffraction data reduction leverages existing capabilities within *DAWN* (Basham *et al.*, 2015 ▸; Filik *et al.*, 2017 ▸) and can be used for detector calibration of the moving beam, data reduction into 2D or 1D profiles, as well as pixel masking, and peak fitting. Automated peak fitting steps using *Jupyter* notebooks can also be included where necessary.

**Figure 11:**
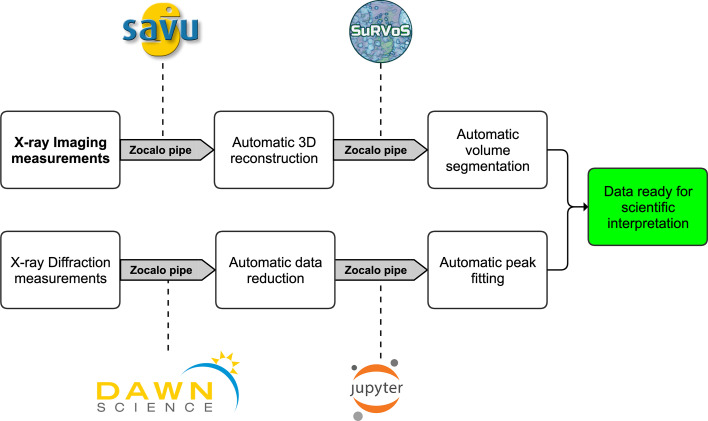
Schematic of DIAD’s data post-processing pipeline, from data collection at the beamline via automated data processing to scientific interpretation.

The aim of this automatic post-processing pipeline is to enable faster scientific interpretation of the experimental results during the experiment as well as high quality and instrument-independent data output ready for eventual publication. The full data processing pipeline is outlined in Fig. 11 ▸.

## 5. Early commissioning results

The beamline provides a tool for the investigation of a wide range of materials, from biomaterials to metals.

The early commissioning experiments, with one biological example and one materials science example, clearly show that the DIAD beamline can be uniquely used to study processes that require simultaneous 3D microstructure and localized strain/stress information.

### 5.1. Example of a biological specimen used for phase identification

Bone implants are used as surgical treatment to provide support during fracture healing as well as replacements for eroded bone. The integrity and long-term stability of the implant is governed by local processes at the bone–implant interface, such as growth of the bone around the implant, as well as local deformation processes and strains/stresses during loading. Synchrotron microtomography, often in combination with digital volume correlation, has so far been used to analyse the deformation processes at the interface (Le Cann *et al.*, 2019 ▸; Madi *et al.*, 2020 ▸). Strains at the bone–implant interface have been investigated using synchrotron diffraction (Mireux *et al.*, 2021 ▸). Combined information from a single specimen, however, is limited. DIAD will be able to provide such multi-modal information.

To mimic a bone implant for commissioning purposes, an implant phantom was prepared. A fractured mouse bone specimen was inserted with an aluminium wire into the medullary cavity of the midshaft. The specimen was wrapped in parafilm to prevent dehydration. Full-field tomography data (pink beam, sample-to-detector distance 65 mm, 3600 projections) and a diffraction map (17 keV, 50 µm × 50 µm beam size, sample-to-detector distance 450 mm) across the full image FOV were collected.

**Figure 12:**
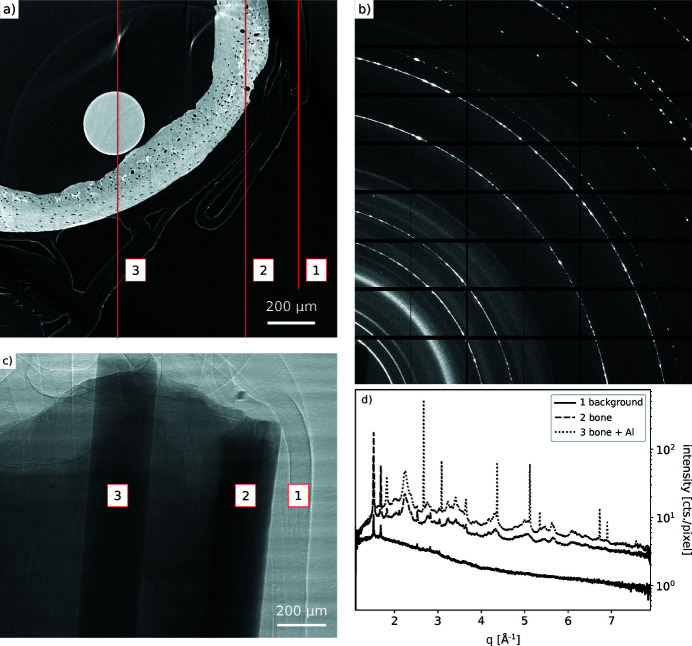
Early results from bone implant commissioning experiment. (*a*) Reconstructed slice from centre of image FOV: bone with lacunae, high- and low-density areas. The vertical lines indicate the locations where diffraction data were taken. (*b*) 2D diffraction pattern of bone and aluminium. (*c*) Radiograph of bone fracture with Al wire. The areas numbered 1, 2 and 3 indicate the regions where the data shown in (*d*) were taken. (*d*) 1D pattern from bone + aluminium, bone and background.

In the reconstructed 3D images [see Fig. 12 ▸(*a*)], cortical bone with the lacunae network is clearly visible. In addition, regions with higher and lower density can be distinguished. Small cracks are also visible. The collected 2D diffraction pattern [see Fig. 12 ▸(*b*)] shows diffuse rings from the bone, and very sharp, textured rings from the aluminium wire.

Combining tomography and diffraction data, the beam path of the diffraction beam through the specimen can be identified. The overlay of both data sets allows the experimentalist to gain an understanding of the origin of the diffraction data [see Fig. 12 ▸(*c*)]. After azimuthal integration, the intensity difference between the two materials becomes even more obvious in the 1D pattern [see Fig. 12 ▸(*d*)]. As a next step, it is envisaged to perform bone implant loading experiments to investigate the area around the bone–implant interface under load conditions.

### 5.2. Example of a metallic specimen used for strain measurement

Copper chrome zirconium alloy (wt%: 1% Cr, 0.1% Zr, CuCrZr) is a candidate material for the cooling pipes in fusion reactors (Alexander *et al.*, 1999 ▸). The loss of reactor cooling due to CuCrZr pipe failure can be catastrophic. It is therefore critical to understand the CuCrZr alloy failure mechanisms. Void nucleation, growth and coalescence have been shown to be prominent ductile failure mechanisms of CuCrZr (Wang *et al.*, 2021 ▸). The distribution of voids and their growth rate, as a function of plastic strain before they coalesce to form a crack, is important information required for modelling the failure of the material, which can be used in the design of the reactor. In application, CuCrZr can be brazed with tungsten by using Cu as a filler material, generating a more complex loading case. For beamline commissioning purposes, the component under study was made of pure Cu as a proof-of-concept.

**Figure 13:**
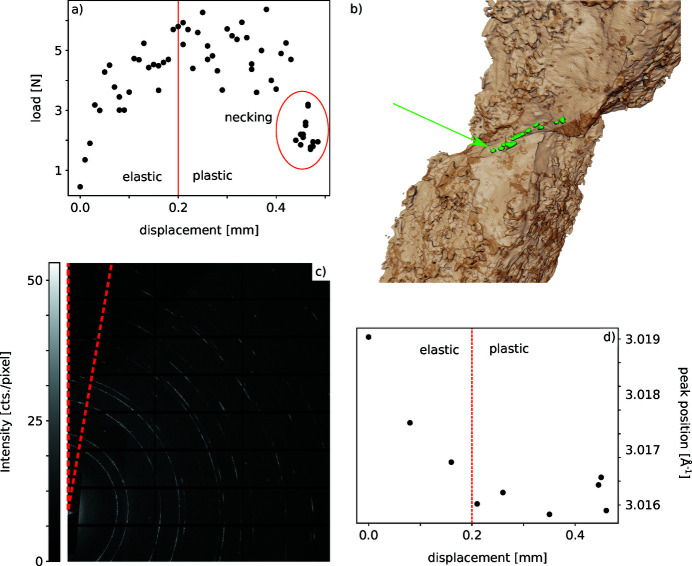
Early results from the CuCrZr deformation commissioning experiment. (*a*) Load–displacement curve: elastic deformation occurs up to 200 µm displacement. (*b*) 3D rendering of plastically deformed CuCrZr; shear bands of voids are present in the necking region in green, highlighted by the green arrow. (*c*) 2D diffraction pattern. The integration was performed over a 10° angle in the loading direction. (*d*) Peak shift of the (311) peak indicating the application of strain in the elastically strained state. The peak position stays constant in the plastic region.

A 150 µm × 250 µm Cu dog bone sample was incrementally loaded to failure in a hand-operated mechanical testing machine. Full-field tomography data (pink beam high angle, 1200 projections, 60 ms per projection) and a diffraction map (29 keV, 50 µm × 50 µm beam size, 5 s exposure time per pattern) were collected at multiple points along the load–displacement curve [see Fig. 13 ▸(*a*)]. It should be noted that the Cu specimen with a 150 µm thickness is at the maximum limit of transmission within the DIAD energy range. The experiment did not use the highest available energy of 37 keV as the diffraction cones on the Pilatus detector were only covering a very small *q*-range and, hence, limited the strain resolution. The incremental application of load to the specimen, which involved manual intervention inside the X-ray hutches as well as the long exposure times required for diffraction data collection from this sample, prevented the use of the beam selector at its high-speed capabilities.

In the tomograms, the first voids with diameter of ∼5 µm become visible once the machine displacement exceeds 200 µm and the material starts to deform plastically. At larger displacements of 400 µm and shortly before specimen failure, necking occurs, and sheer bands of voids can be found [see Fig. 13 ▸(*b*)].

Diffraction data were collected to monitor peak shifts and, consequently, strains within the material as it was loaded. The 2D diffraction pattern [see Fig. 13 ▸(*c*)] was radially integrated over a 10° angle in the direction of the applied load. The positions of the Cu (311) peak were plotted [see Fig. 13 ▸(*d*)]. A displacement increase in the elastic region causes a peak shift to lower *q*-values, whereas in the plastic region the peak position remains unchanged.

The presented results of tomography and diffraction are in line with expectation and indicate that, at DIAD, it is entirely possible to resolve particles and voids a few micrometres in size from micro-computed tomography while simultaneously measuring strains.

## 6. Facility access

The DIAD beamline is accessible through the standard Diamond peer-reviewed proposal system. Beam time can also be requested through the Long-Term Proposal procedure. Finally, beam time can also be obtained on a commercial basis by contacting the Diamond Industrial Liaison Office.

## 7. Summary

We have presented here the new DIAD (Dual Imaging and Diffraction) beamline at Diamond. The instrument has two independent beams that meet at the sample position and combines two analysis techniques: 2D/3D microstructural information can be obtained from imaging and tomography; phase and/or strain information from diffraction. Both techniques can be switched at a frequency to enable simultaneous acquisition of imaging and powder diffraction from a sample during a process.

Features of the beamline include multiple specimen manipulation environments that set the beamline up for *in situ* and *in operando* as well as time-resolved measurements.

Advanced data acquisition strategies as well as automated data processing pipelines aid the user during the decision-making process and live data analysis.

The beamline enables new types of microstructure and composition/strain analysis that so far could not be done easily on already existing instruments.

## Figures and Tables

**Table 1:** DIAD beamline optic and key operation parameters

Beamline optic	Diffraction branch	Imaging branch
Source	Ten-pole wiggler as common source for both branches	
Mirrors operation mode	M1 flat mirror	M3 flat mirror
	M2 bendable mirror	M4 bendable mirror
	Monochromatic	Pink or monochromatic
Slits	S2 slits for DCM off-set compensation	S3 for DCM off-set compensation and beam size changes
Monochromators	DCM1 with Si (111) crystals	DCM2 with Si (111) crystals
Beam selector	Beam selector shared between both branches	
Focusing elements	Platinum-coated KB system	Not applicable
Maximum beam size at sample position	Variable beam size between 13 µm × 4 µm up to 50 µm × 50 µm	1.7 mm × 1.7 mm

**Table 2:** Sample manipulation stages at the DIAD beamline

	General tomography stage	Platform stage
Available motion	Vertical motion (50 mm)	Vertical motion (50 mm)
	Rotation stage for tomography	Horizontal motion for sample alignment in the field of view (50 mm each direction)
	Horizontal motion for sample alignment in the field of view (5 mm each direction)	
Maximum specimen weight	10 kg for slow speed	100 kg
	500 g for high speed (>3 Hz)	

**Table 3:** Experimental protocol: the type of trigger and threshold need to be specified together with the type of X-ray measurement that will be triggered

Trigger	Threshold	X-ray measurement
Discharge = 0 mA h	0	Diffraction
Voltage = 3.9 V	±0.01	Diffraction
Voltage = 3.5	±0.05	Tomography
Discharge = 100 mA h	±2	Diffraction
Voltage < 3 V	Not applicable	Tomography
Discharge = 300 mA h	±2	Diffraction
Discharge = 325 mA h	±2	Diffraction
